# Predictors of Parent-Reported Health-Related Quality of Life in Young Children with Early Brain Damage and Severe Motor Dysfunction

**DOI:** 10.3390/jcm14197054

**Published:** 2025-10-06

**Authors:** Siri Johnsen, Kristian Sørensen, Jon Sverre Skranes, Ida Eline Vestrheim, Mette Gro Modahl, Reidun Birgitta Jahnsen, Kristine Stadskleiv, Gry Hansen, Stian Lydersen, Rannei Sæther

**Affiliations:** 1 Department for Clinical and Molecular Medicine, Faculty of Medicine and Health Sciences, Norwegian University of Science and Technology, 7113 Trondheim, Norway; kristian.sorensen@sshf.no; 2 Departments of Children and Youth, Arendal and Kristiansand, Sørlandet Hospital HF, 4604 Kristiansand, Norway; jon.skranes@sshf.no (J.S.S.); ida.vestrheim@sshf.no (I.E.V.); mette.modahl@sshf.no (M.G.M.); 3 Department of Research, Beitostølen Healthsports Center, 2953 Beitostølen, Norway; reijah@ous-hf.no; 4 Department of Public Health and interdisciplinary Health Sciences, University of Oslo, 0317 Oslo, Norway; 5 NorCP, Department of Clinical Neurosciences for Children, Oslo University Hospital, 0424 Oslo, Norway; kristine.stadskleiv@isp.uio.no; 6 Department of Special Needs Education, University of Oslo, 0318 Oslo, Norway; 7 Department of Habilitation for Children and Youth, St. Olav Hospital HF, 7006 Trondheim, Norway; gry.hansen@stolav.no; 8 Regional Centre for Child and Youth Mental Health and Child Welfare, Department of Mental Health Faculty of Medicine and Health Sciences, Norwegian University of Science and Technology, 7130 Trondheim, Norway; stian.lydersen@ntnu.no (S.L.); rannei.sather@ntnu.no (R.S.); 9 Department of Rehabilitation Science and Health Technology, Faculty of Health Sciences, OsloMet, 0130 Oslo, Norway

**Keywords:** children, severe disabilities, HRQOL, health status and well-being, adaptive functions, gross motor function, communication functions, intensive habilitation program

## Abstract

**Background/Objectives**: This cross-sectional study aimed to identify predictors of parent-reported health-related quality of life (HRQOL) in young children with early brain damage and severe motor dysfunction. It used baseline data from the PIH Multi Study, a randomized controlled trial evaluating an intensive, family-centered habilitation program for preschool children and their parents. **Methods**: Parent-reported HRQOL were measured using the CPCHILD questionnaire. Potential predictors included adaptive function (PEDI-CAT), gross motor function (GMFM-66), postural control and balance (ECAB), and communication function (FOCUS). These were selected to reflect the domains of the ICF-CY framework. Data were collected by professionals and by parents. Linear regression analyses were conducted to identify significant predictors. **Results**: Analyses included 65 children. Better adaptive skills, gross motor function, postural control, and communication all predicted higher parent-reported HRQOL. Adaptive skills—particularly in self-care and mobility—and gross motor function emerged as the strongest predictors. **Conclusions**: The study highlights the importance of targeting basic functional skills in early habilitation efforts for children with severe disabilities. The findings support a multidimensional understanding of health in line with the ICF-CY framework and underline the value of early, individualized, and family-centered interventions. Future research should investigate these predictors longitudinally and explore ways to integrate children’s own perspectives in assessment of HRQOL.

## 1. Introduction

The concept of health has evolved significantly in recent decades, moving beyond the traditional biomedical model to a more holistic understanding that includes physical, psychological, and social well-being [1]. This broader perspective is reflected in the increasing use of health-related quality of life (HRQOL) as an outcome measure for children with complex, long-term health conditions [2,3].

HRQOL refers to how an individual’s health status influences their perceived quality of life across various domains, including physical functioning, emotional well-being, independence, and social relationships [4] (pp. 503–504). For young children under school age, self-reporting on questionnaires is not feasible. In such cases, caregiver-reported outcomes become essential for capturing aspects of HRQOL in daily life [3,5].

Children with early brain damage and severe motor dysfunction, including cerebral palsy (CP), often experience a mix of motor impairments, pain, communication barriers, cognitive challenges, and limited participation opportunities [2,6]. These difficulties can greatly affect both daily functioning and overall quality of life [7,8]. However, understanding of which specific functional abilities are the strongest predictors of HRQOL in this group remains limited, particularly during early childhood.

To address this knowledge gap, this study draws on the framework of the International Classification of Functioning, Disability and Health—Children and Youth version (ICF-CY) [9]. The ICF-CY provides a biopsychosocial model for understanding health and functioning, emphasizing the interaction between body functions, activities, participation, and environmental factors. This multidimensional view of health highlights the importance of assessing function across multiple domains when evaluating HRQOL in children with disabilities [4].

Studies have found associations between HRQOL and motor function, cognitive and communication impairments, pain, emotional health, participation, and family support [2,7,8,10]. Other studies have shown association between hip displacement and recurrent musculoskeletal pain with reduced HRQOL in children with CP [11,12]. However, few studies have systematically examined which of these domains in young children with severe motor impairment are the strongest predictors of their HRQOL.

The aim of this study was to examine how different domains of functioning predict HRQOL in young children with early brain damage and severe motor dysfunction. Specifically, the study explored the extent to which adaptive behavior, gross motor skills, balance, and communication abilities predict caregiver-reported HRQOL. By identifying which functional domains were the strongest predictors of HRQOL, the study sought to strengthen the knowledgebase needed to guide early intervention and the development of family-centered habilitation services for these children.

## 2. Materials and Methods

### 2.1. Study Design

This cross-sectional study utilized baseline data from the PIH Multi Study, an ongoing multi-center randomized controlled trial (RCT) with a stepped-wedge design, which investigates the effects of a program of intensive habilitation (PIH) on adaptive, communicative, and gross motor functions in preschool children with early brain damage and severe motor dysfunction in Norway [13,14].

The PIH Multi Program, developed at Sørlandet Hospital in Norway, is a family-centered, multidisciplinary initiative. The program spans approximately one year and includes three in-patient group sessions, each lasting two weeks. Between these sessions, the children participate in individualized functional training programs integrated into their daily routines and activities at home and in kindergarten [15].

### 2.2. Recruitment and Evaluation

Participants were recruited from all Health Regions in Norway by key personnel in local pediatric habilitation departments and collaborators at Regional University Hospitals.

Following recruitment, baseline evaluations were conducted by three independent clinicians with extensive experience from pediatric habilitation departments. Two physiotherapists performed the motor assessments, while a special education teacher carried out the communication assessments. Parents received questionnaires by mail and were asked to return the completed forms to the physiotherapist assessing their child. Baseline data collection took place between September 2021 and September 2024. 

### 2.3. Participants

All children referred to the PIH Multi program who met the inclusion criteria were offered participation in the study. To be eligible, children had to be aged 2–7 years of age when entering the program and they had to have a diagnosis of severe CP or a similar diagnosis with Gross Motor Function Classification System (GMFCS) levels III, IV or V. Ambulatory status was defined based on GMFCS level, where children classified at level III were considered ambulant, and those at levels IV and V were considered non-ambulant. Additionally, the recruited children needed to be capable of participating in group sessions, and their parents had to be willing to learn and actively engage in their child’s training. At least one parent was required to speak fluent Norwegian or English. Exclusion criteria included progressive neurologic disorders or comorbidities such as autism spectrum disorder, severe visual and hearing impairments, or intractable epilepsy.

### 2.4. Measures

Caregiver Priorities and Child Health Index of Life with Disabilities (CPCHILD) is a parent reported questionnaire designed to assess HRQOL in terms of health status, comfort and well-being, and ease of caregiving for children with severe CP, aged 5 to 18 years [16]. This HRQOL measure is available in both proxy versions and self-report for older children. The CPCHILD consists of six subdomains: 1. Personal care and activities of daily living, 2. Positioning, transferring and mobility, 3. Comfort and emotions, 4. Communication and social interaction, 5. Health, and 6. Overall quality of life. Good validity and reliability of the Scandinavian version is established [17]. The scores are standardized for each domain and for the total survey, ranging from 0 (worst) to 100 (best) [5]. Children classified at GMFCS level III typically have CPCHILD total scores around 70, while children at GMFCS level V score closer to 45 [7].

Pediatric Evaluation of Disability Inventory—Computer Adaptive Test (PEDI-CAT) is an online caregiver report that measures functional skills in children and youth, aged 2 to 20 years [18]. There are four domains: self-care, mobility, social/cognitive, and responsibility. Reliability and construct validity have been established for children with medical complexity [19]. The PEDI-CAT has been translated into Norwegian in 2017 [20]. An analysis of structural validity indicated that the Norwegian version of PEDI-CAT had acceptable reliability to measure degree of functioning or responsibility [21]. Each domain is scored separately, with scaled scores up to 100 points, and standardized scores adjusted for age [18] (pp. 648–657).

Gross Motor Function Measure 66 (GMFM-66) is a clinical test developed to measure gross motor function in cerebral palsy, age range 5 months to 16 years [22,23]. The measure covers gross motor function in lying/rolling, sitting, crawling/kneeling, standing and walking/running/jumping. Good reliability and validity are established [23]. More recent studies have used GMFM-66 to evaluate gross motor function in young children with non-progressive neurodevelopmental disorders [24]. The score is a scaled score representing the child’s gross motor function on a scale from 0 (lowest) to 100 points (highest).

Early Clinical Assessment of Balance (ECAB) is a clinical test developed to measure postural control and balance in young children with CP, aged 18 months to 5 years [25]. The ECAB consists of two subscales: Part I assesses postural control in head and trunk, while Part II evaluates postural control in sitting and standing. Developmental trajectories were outlined to indicate prognoses [26]. Validity and reliability are established [25,27]. The maximum score for Part I is 36 points and for Part II 64 points, leading to a total possible score of 100 points. Higher scores indicate better postural control and balance [25].

Focus on the Outcomes of Communication, Under Six (FOCUS)—34 is a structured interview of parents designed to evaluate treatment change concerning communicative participation in young children, aged 18 months to 6 years [28]. Communicative participation refers to the child’s communication and interaction in “real world” situations at home, in school, or in the community [29]. Construct validity was established by Washington at al. (2013) [30]. The questionnaire is translated into Norwegian [31], but not yet standardized in a northern European setting. The maximum total score is 238 points, with a starting point at 34 points [26].

The Children’s department of Sørlandet Hospital’s self-report questionnaire was employed to collect background information on the children and parents. The questionnaire also included questions about any additional impairments the child may have, beyond motor dysfunction, as reported by the parents. Background characteristics on parents were collected according to Hollingshead Socioeconomic Status [32].

### 2.5. The ICF-CY Framework and Selection of Outcome Measures

**Figure 1:**
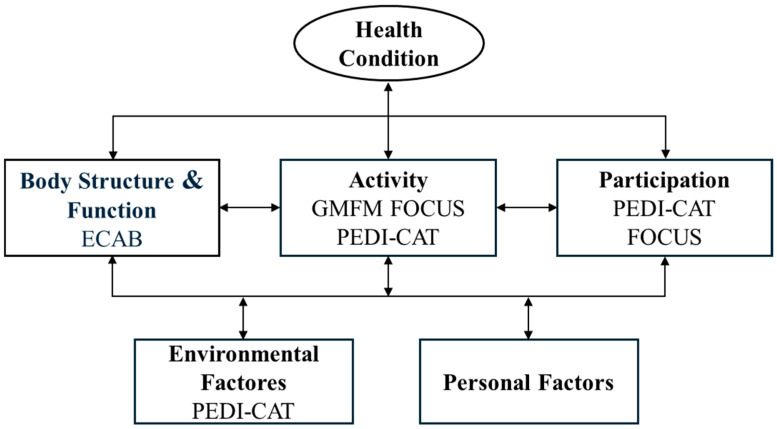
Overview of the ICF -CY framework and the predictors of HRQOL.

To reflect the multidimensional nature of functioning described in the ICF framework, this study included outcome measures representing different domains in the model. As illustrated in Figure 1, the ICF-CY distinguishes between body functions, activities, and participation, all influenced by environmental and personal factors. The ECAB primarily reflects body functions. The GMFM, PEDI-CAT, and FOCUS are mainly associated with the activity domain [28,33,34], while PEDI-CAT and FOCUS also relate to participation [28,34]. In addition, PEDI-CAT includes items reflecting environmental factors.

CPCHILD is a measure of HRQOL related to the framework by providing a complementary perspective on perceived health and well-being within its components [35].

### 2.6. Statistical Analysis

Descriptive statistics were calculated for the CPCHILD, PEDI-CAT, GMFM, ECAB and FOCUS. This included measures of central tendency (mean) and dispersion (minimum, maximum, range, and standard deviation).

Missing data for CPCHILD were managed following the guidelines of Petterson et al., which recommend that parents must answer at least 80% of all subdomains and or the total of 37 questions [17]. Questionnaires with more than 20% missing responses were excluded from the analysis. Additionally, an attrition analysis was performed to assess any systematic differences between participants with complete and incomplete data.

We used linear regression analyses with CPCHILD as the dependent variable. The main predictor variables were adaptive function (measured with PEDI-CAT), gross motor function (measured with GMFM-66), postural control and balance (measured with ECAB) and communicative participation (measured with FOCUS). Each predictor variable was examined separately, unadjusted, and adjusted for the child’s age and ambulatory status (walking or non-walking). The same approach was applied to the remaining research questions, with GMFM-66, ECAB total score, and FOCUS total score serving as the primary predictor variable in their respective models.

To compare the predictive value of the various measures and to understand the clinical significance of the predictions, the regression coefficient was multiplied by the standard deviation (SD) of the predictor variable to estimate its effect on CPCHILD total score. This approach was used to determine whether the predicted effect exceeded the minimal clinically important difference (MCID) for CPCHILD, which have been estimated to range between 5 to 9 points [2].

Normality of the residuals was confirmed by visual inspection of Q-Q plots. Ninety-five percent confidence intervals (CI) are reported where relevant.

### 2.7. Ethics

This study was conducted in accordance with the Helsinki declaration. Regional Committee for Medical and Health Research Ethics has given their approval (Reference number 228805 and 11 August 2021).

### 2.8. Use of GenAI in Writing

During the preparation of this manuscript, the authors used SIKT Copilot, a generative AI-based tool, to improve language and phrasing. The tool was only employed for text refinement. All scientific content, data interpretation, and conclusions were produced and verified by the authors.

## 3. Results

### 3.1. Participants

**Figure 2:**
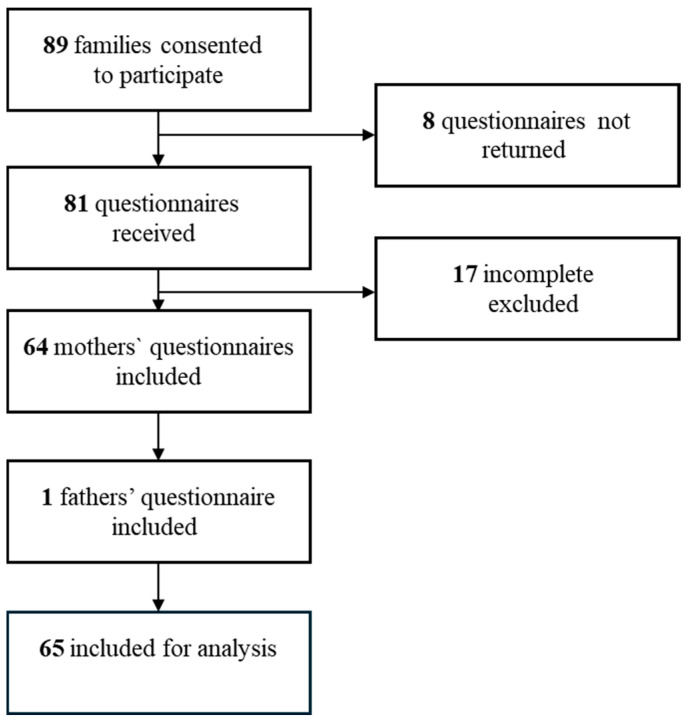
Inclusion and exclusion of CPCHILD questionnaires for analysis.

A total of 89 families consented to participate in the study. Details are presented in the flowchart (Figure 2), which also illustrates missing responses on the primary outcome measure, the CPCHILD questionnaire. Eight did not return the questionnaire, and an additional 17 were excluded due to more than 20% missing data. This resulted in 65 completed questionnaires being included in the analysis. Both parents were invited to complete the CPCHILD, but in most cases, the mother provided the most complete response. In 64 cases, the mother’s questionnaire was used. In one case, where the mother was not present, the father’s completed questionnaire was included.

The sample of 65 consisted of 32 girls and 33 boys with a mean age of 45.6 months (Table 1). More than half of the children had CP, while the others had severe developmental delay (of unknown cause) or a genetic disorder as their primary diagnosis. Thirteen of the children were ambulant (walking), whereas 52 (80.0%) were non-ambulant (non-walking). The most frequently reported impairments, in addition to motor difficulties, included communication (83%), nutrition (59%) and cognition (51%).

The mean age of mothers was 34.6 years, 68% of the parents in the study sample had higher education, and 14% of the mothers were born outside Norway, Table 1.

### 3.2. Clinical Characteristics

Table 2 presents the clinical characteristics of the 65 participants. The assessment results showed that the children had extensive functional limitations. Most required considerable assistance in daily activities related to mobility, self-care, social-cognitive functioning, and responsibility. Gross motor abilities were primarily limited to movements in lying and supported sitting positions, with no observed ability to crawl, stand, or walk. Postural control and balance were also limited, with adequate control mainly in lying positions and reduced balance while sitting. Communication abilities varied but were generally limited across the sample. The range of scores indicated variability in functional levels among the participants.

Attrition analyses revealed that the group of 24 excluded children (Figure 2) had substantially lower mean scores on both the GMFM-66 and the FOCUS total scores compared to the study group, with differences of 9.4 and 19.1 points, respectively (see Appendix A.1). These differences exceeded the described MCID thresholds, which range from 1.5 to 3.7 for the GMFM-66 [36] and 16 for the FOCUS [37]. The mean scores of PEDI-CAT and ECAB did not differ substantially between the included and excluded groups.

### 3.3. Regression Analyses

Results of linear regression analyses with one covariate at a time, are reported in Table 3. Results adjusted for age and ambulatory status gave similar results as the unadjusted models, reported in Appendix A.2 The results showed that adaptive skills, gross motor function, postural control and balance, and communication function were significant predictors of HRQOL. Positive coefficients indicated that higher functional levels in these areas were associated with better outcomes on the CPCHILD.

#### 3.3.1. Adaptive Skills

All four subdomains of the PEDI-CAT were significant predictors of the CPCHILD scores. One SD difference in the Personal Care subdomain (6.0 points) predicted a 6.90-point increase in CPCHILD scores. One SD difference in the Mobility subdomain (7.0 points) predicted a 6.79-point increase in CPCHILD scores. One standard deviation difference in the Social/Cognitive subdomain (7.1 points), predicted 4.83-point increase in CPCHILD scores. One standard deviation difference in the Responsibility subdomain (7.0 points) predicted 5.81-point increase in CPCHILD score.

#### 3.3.2. Gross Motor Function

Gross motor function and postural control were also significant predictors of health status and well-being. One SD difference in GMFM-66 (11.9 points) predicted a 6.43-point increase in CPCHILD scores. One standard deviation difference in ECAB total score (10.7 points) predicted 4.82-point increase in CPCHILD score.

#### 3.3.3. Communicative Abilities

A significant prediction was also observed between communication abilities and CPCHILD scores. One SD difference in FOCUS total scores (47.1 points) predicted a 5.65-point increase in CPCHILD scores.

## 4. Discussion

This study found that parent-reported HRQOL was significantly predicted by several domains of functioning in young children with early brain damage and severe motor dysfunction. Specifically, better adaptive skills, gross motor function, postural control, and communication skills all predicted higher scores on the CPCHILD. Among these, adaptive skills, particularly in self-care and mobility, as well as gross motor function were identified as the strongest predictors. These findings underscore the relevance of basic functional abilities in shaping perceived HRQOL in this vulnerable population.

### 4.1. Interpretation of Findings in Relation to Previous Research

#### 4.1.1. Adaptive Skills

The finding that adaptive skills strongly predict HRQOL aligns with previous studies emphasizing the importance of everyday functioning in shaping well-being of children with disabilities [8]. In this study, adaptive skills related to personal care and mobility contributed most substantially to parent-reported HRQOL, suggesting that children’s ability to participate in basic daily routines may play a key role in how caregivers perceive their HRQOL

These abilities are central to independence, reduce caregiver burden, and may enhance the child’s experience of autonomy and dignity, even when functioning is supported or assisted. From an ICF-CY perspective, adaptive skills bridge the domains of activity and participation, and their relevance may reflect the close interdependence of functioning and everyday life quality in early childhood.

#### 4.1.2. Gross Motor Function and Postural Control

Gross motor functioning and postural stability were also significant predictors of HRQOL. This aligns with earlier research indicating that children with better motor function tend to have higher quality of life, particularly through improved mobility and greater opportunities for participation [2,7,10]. Improved gross motor ability may facilitate not only physical access to the environment but also active engagement in play, interaction and exploration, all activities essential for development and well-being in early childhood.

Postural control, often an overlooked domain [7,8], may in the ICF-CY perspective contribute to comfort, safety, and interactional competence in everyday settings, particularly for children who rely on assistive technology or alternative positioning. Its predictive value in this study suggests that even basic physical functions can influence broader perceptions of HRQOL.

#### 4.1.3. Communicative Abilities

Communication abilities also significantly predicted parent-reported HRQOL. Although not as strong a predictor as adaptive skills or gross motor function, the findings suggest that both verbal and nonverbal communication play an important role in how children’s quality of life is perceived by the parents. This finding supports the central role of communicative abilities in enabling children to express themselves during social interactions and participation in daily routines [33]. Even limited or assisted communication can contribute to more meaningful interactions between children and their caregivers, potentially influencing how caregivers evaluate the child’s overall well-being.

From an ICF-CY perspective, communication intersects with both activity and participation domains. The inclusion of communication as a predictor highlights the importance of supporting communicative competence in habilitation efforts, especially in children with motor impairments who may be at risk of isolation, unmet participation opportunities and needs due to limited expressive abilities.

### 4.2. Underlying Mechanisms and Interpretations

Together, these findings point to the relevance of functional abilities that support independence, activity, and participation in everyday life. According to the ICF-CY framework, impairments in body function can lead to limitations in activity and restrictions in participation, which in turn can influence HRQOL. Our results support this model, suggesting that when children are more capable of participating in basic routines, whether through independent or supported movement, postural control, or communication, caregivers perceive their child’s HRQOL more positively.

This may also reflect the impact of reduced caregiver burden, increased shared experiences, and a stronger sense of child–parent reciprocity when functional abilities are higher [2]. It is important to acknowledge that the interpretation of HRQOL in this study is based on proxy reports and thus reflects the caregivers’ lens on the child’s daily life and health status.

### 4.3. Strengths and Limitations

Several methodological strengths and limitations should be considered when interpreting the findings. A strength of the study is the inclusion of multiple validated outcome measures covering different functional domains, aligned with the ICF-CY framework. In addition, the use of CPCHILD provides a structured and relevant proxy measure of HRQOL for children with severe impairments.

Although CPCHILD is validated for individuals aged 5 to 18 years, other studies have included younger children [38,39,40,41]. There are few alternative HRQOL assessment options available for young children with severe disabilities. Therefore, we consider CPCHILD appropriate for assessing HRQOL in this population, even though the mean age is lower than five years.

Certain limitations should be acknowledged. Information about the etiology of brain damage was not collected due to ethical constraints in the original study approval. Additionally, the study did not include a control group with non-congenital brain damage, and subgroup analyses beyond ambulatory status were not feasible due to limited sample size. These factors may restrict generalizability and should be considered in future research designs.

The cross-sectional design limits causal interpretation. It is not possible to determine whether higher function leads to better perceived quality of life, or whether children with better general health are more capable of functioning across domains. Furthermore, relying solely on parent-reports may introduce bias, such as caregiver well-being, expectations, and experiences may shape their perceptions of the child’s quality of life.

The sample represents a specific clinical population, and results may not be generalized to children with milder impairments or different contextual factors. A potential risk of selection bias should be considered, as the most severely impaired children were less represented in the included group than in the excluded group. However, the mean CPCHILD score in our sample was 54.4, aligning with the typical range for children at GMFCS levels III to V, and indicating that our cohort is representative of this severity of gross motor impairment

Future studies could benefit from triangulating caregiver reports with clinical observations, and when possible, incorporating the child’s own voice through adapted self-report tools or qualitative methods.

### 4.4. Clinical Implications and Future Research

The findings underscored the importance of targeting adaptive skills and motor function in habilitation for children with early brain damage and severe motor dysfunction. Targeting gross motor function and self-care, communication, and posture may contribute not only to functional outcomes but also to improved HRQOL as perceived by parents. Early, multidisciplinary, and family-centered approaches remain crucial in addressing the complex needs of this population.

Future research should investigate these predictors longitudinally to better under-stand how development, functioning, and perceived well-being change over time. To broaden the scope, future studies may include additional functional areas such as hand and fine motor function—as potential predictors. Greater emphasis should also be placed on contextual factors, including family support, access to professionals with expertise in supported physical activity and assisted personal care, availability of assistive technology and devices, and opportunities for participation—factors that interact with functional abilities to shape HRQOL.

## 5. Conclusions

This study found that adaptive skills, gross motor function, postural control, and communication significantly predicted parent-reported HRQOL in young children with early brain damage and severe motor dysfunction. Adaptive skills and gross motor function emerged as the strongest predictors, emphasizing the importance of everyday functioning for quality of life.

These findings support a multidimensional understanding of health, as described in the ICF, and highlight the need for early, targeted interventions that address key areas of function. Future research should explore these predictions longitudinally and consider incorporating children’s own perspectives.

## Figures and Tables

**Table 1:** Demographic characteristics of children and parents.

Children n = 65		
**Age in months**	Mean (SD)	45.6 (16.8)
	Min/max	15.8/83.5
**Sex**	Boys	33 (51)
	Girls	32 (49)
**Diagnosis**	CP	35 (54)
	Other ^(a)^	30 (46)
**Mobility**	Ambulant	13 (20)
	Non-ambulant	52 (80)
**Child impairments, as reported by parents ^(b)^**	Epilepsy	22 (34)
	Vision	24 (37)
	Hearing	7 (11)
	Respiration	18 (28)
	Communication	54 (83)
	Cognition	33 (51)
	Nutrition	38 (59)
	Sleep	26 (40.0)
	Pain	6 (9)
**Parents n = 65**		
**Age**	Mean (SD)	34.6 (5.6)
**Education ^(c)^**	≤10 years	1 (2)
	11–13 years	19 (29)
	Diploma/ Bachelor	21 (32)
	Master	20 (31)
	Doctoral degree	4 (6)
**Employment ^(d)^**	Adm. Management	1
	Academics	15
	Technicians, lower academics	4
	Sales, service, office, care	33
	Farming, forestry, fishing, craftsmen transport	3
	Student/childcare	11
	Other	4
**Born outside Norway**		9

Data are n (%) unless otherwise stated. CP; Cerebral Palsy ^(a)^ Other diagnosis includes developmental delay, and genetic disorders, ^(b)^ Parents reported impairments extracted from the background questionnaire is more than 65 because the parents reported more than one impearment ^(c)^ Level of education, ^(d)^ Type of employment, Employment did not add up to 65 because 6 have registered two categories of employment.

**Table 2:** Clinical characteristics.

Measurement	N	Mean (SD) **	Min–Max
CPCHILD Total score	65	54.4 (11.9)	26.7–83.3
PEDI-CAT Self-care	65	42.3 (6.0)	30.0–56.0
PEDI-CAT Mobility	65	47.0 (7.0)	33.0–63.0
PEDI-CAT Social/Cognitive	65	52.8 (7.1)	38.0–66.0
PEDI-CAT Responsibility	41 *	34.1 (7.0)	25.0–49.0
GMFM-66 Total score	65	34.9 (11.9)	10.4–65.4
ECAB Total score	65	25.5 (10.7)	4.0–44.0
FOCUS Total score	65	102.8 (47.1)	52.5–228.0

* N = 41 for this variable due to age > 3 years for the responsibility domain. PEDI-CAT; Paediatric Evaluation of Disability Inventory Computer Adaptive Test, GMFM-66; Gross Motor Function Measure 66, EACB; Early Clinical Assessment of Balance, FOCUS; Focus on the Outcomes of Communication Under Six. ** In all measures, higher scores indicate better function. Possible ranges of scores for CPCHILD Total score 0–100; PEDI-CAT 0–100; GMFM-66 0–100; ECAB 0–100; FOCUS 34–238.

**Table 3:** Linear regression with CPCHILD as dependent variable.

Covariate	Regression Coefficient (Estimate and CI)	*p*-Value	Predicted Change * (Estimate and CI)
PEDI-CAT Self-care	1.15 (0.75–1.56)	<0.001	6.90 (4.50–9.36)
PEDI-CAT Mobility	0.97 (0.62–1.32)	<0.001	6.79 (4.34–9.24)
PEDI-CAT Social/Cognitive	0.68 (0.20–1.07)	<0.001	4.83 (4.50–9.24)
PEDI-CAT Responsibility	0.83 (0.29–1.37)	0.004	5.81 (2.13–7.60)
GMFM-66 Total score	0.54 (0.33–0.75)	<0.001	6.43 (3.93–8.93)
ECAB Total score	0.45 (0.21–0.70)	<0.001	4.82 (2.25–7.49)
FOCUS Total score	0.12 (0.07–0.17)	<0.001	5.65 (2.82–8.01)

* Predicted change per SD of the covariate. PEDI-CAT; Pediatric Evaluation of Disability Inventory Computer Adaptive Test, GMFM-66; Gross Motor Function Measure 66, EACB; Early Clinical Assessment of Balance, FOCUS; Focus on the Outcomes of Communication Under Six.

## Data Availability

The data presented in this study may to some extent be available on request from the corresponding author. The data is not publicly available due to ethical reasons, since public sharing of data was not specifically consented for by participants.

## Appendix A

### Appendix A.1

**Table A1:** Attrition analysis.

Measurement	Included Total N = 65	Excluded Total N = 24
CPCHILD Total score	65 54.4 (11.9)	
PEDI-CAT Self-care	65 42.2 (6.0)	23 41.6 (4.5)
PED-ICAT Mobility	65 47.0 (7.0)	23 48.0 (5.3)
PEDI-CAT Soc/cog	65 52.8 (7.1)	23 51.2 (5.4)
PEDI-CAT Responsibility	56 33.1 (7.0)	15 31.4 (5.4)
FOCUS Total score	65 102.8 (47.1)	23 83.7 (27.26)
GMFM-66 score	65 34.9 (11.9)	24 25.5 (10.7)
Age	65 45.6 (16.8)	24 46.1 (12.5)
Non-ambulant	53/65 (81.5)	21/24 (87.5)

Data are n, mean (SD) or n/N (%). CPCHILD; Caregiver Priorities and Child Health Index of Life with Disabilities, PEDI-CAT; Paediatric Evaluation of Disability Inventory Computer Adaptive Test, GMFM-66; Gross Motor Function Measure 66, EACB; Early Clinical Assessment of Balance, FOCUS; Focus on the Outcomes of Communication Under Six.

### Appendix A.2

Linear regression with CPHILD as dependent variable, one covariate at the time adjusted for age and ambulatory status.

**Table A2:** Linear regression analysis, unadjusted and adjusted for age, ambulant and both.

Regression Coefficient				
Covariate	N	Estimate	95% CI	*p*-Value
PEDI-CAT Self-care				
Unadjusted	65	1.15	0.75–1.56	<0.001
Adjusted for				
Age	65	1.22	0.80–1.64	<0.001
Ambulant	65	1.09	0.65–1.63	<0.001
Age and ambulant	65	1.11	0.70–1.61	<0.001
PEDI-CAT Mobility				
Unadjusted	65	0.97	0.62–1.32	<0.001
Adjusted for				
Age	65	1.02	0.66–1.38	<0.001
Ambulant	65	1.03	0.60–1.66	<0.001
Age and ambulant	65	1.07	0.63–1.52	<0.001
PEDI-CAT Social/Cognitive				
Unadjusted	65	0.68	0.30–1.07	<0.001
Adjusted for				
Age	65	0.71	0.31–1.12	<0.001
Ambulant	65	0.59	0.20–0.99	0.004
Age and ambulant	65	0.62	0.21–1.04	0.004
PEDI-CAT Responsibility				
Unadjusted	41	0.83	0.29–1.37	0.004
Adjusted for				
Age	41	0.83	0.28–1.38	0.004
Ambulant	41	0.75	0.19–1.31	0.011
Age and ambulant	41	0.75	0.17–1.33	0.013
FOCUS total score				
Unadjusted	65	0.16	0.06–0.17	<0.001
Adjusted for				
Age	65	0.16	0.06–0.17	<0.001
Ambulant	65	0.11	0.05–0.16	<0.001
Age and ambulant	65	0.11	0.05–0.17	<0.001
GMFM-66				
Unadjusted	65	0.54	0.33–0.75	<0.001
Adjusted for				
Age	65	0.55	0.34–0.77	<0.001
Ambulant	65	0.60	0.32–0.87	<0.001
Age and ambulant	65	0.61	0.32–0.89	<0.001
ECAB Total score				
Unadjusted	65	0.45	0.21–0.70	<0.001
Adjusted for				
Age	65	0.46	0.21–0.71	<0.001
Ambulant	65	0.39	0.11–0.68	0.008
Age and ambulant	65	0.40	0.11–0.69	0.008

PEDI-CAT; Paediatric Evaluation of Disability Inventory Computer Adaptive Test, GMFM-66; Gross Motor Function Measure 66, EACB; Early Clinical Assessment of Balance, FOCUS; Focus on the Outcomes of Communication Under Six.
